# Synthesis of Glycopolymer Micelles for Antibiotic Delivery

**DOI:** 10.3390/molecules28104031

**Published:** 2023-05-11

**Authors:** Xuan Chen, Bin Wu, Harini A. Perera, Mingdi Yan

**Affiliations:** Department of Chemistry, University of Massachusetts Lowell, Lowell, MA 01854, USA

**Keywords:** glycopolymer, poly(lactic acid), carbohydrate, drug delivery, antibiotics

## Abstract

In this work, we designed biodegradable glycopolymers consisting of a carbohydrate conjugated to a biodegradable polymer, poly(lactic acid) (PLA), through a poly(ethylene glycol) (PEG) linker. The glycopolymers were synthesized by coupling alkyne end-functionalized PEG-PLA with azide-derivatized mannose, trehalose, or maltoheptaose via the click reaction. The coupling yield was in the range of 40–50% and was independent of the size of the carbohydrate. The resulting glycopolymers were able to form micelles with the hydrophobic PLA in the core and the carbohydrates on the surface, as confirmed by binding with the lectin Concanavalin A. The glycomicelles were ~30 nm in diameter with low size dispersity. The glycomicelles were able to encapsulate both non-polar (rifampicin) and polar (ciprofloxacin) antibiotics. Rifampicin-encapsulated micelles were much smaller (27–32 nm) compared to the ciprofloxacin-encapsulated micelles (~417 nm). Moreover, more rifampicin was loaded into the glycomicelles (66–80 μg/mg, 7–8%) than ciprofloxacin (1.2–2.5 μg/mg, 0.1–0.2%). Despite the low loading, the antibiotic-encapsulated glycomicelles were at least as active or 2–4 times more active than the free antibiotics. For glycopolymers without the PEG linker, the antibiotics encapsulated in micelles were 2–6 times worse than the free antibiotics.

## 1. Introduction

Polymeric micelles are among the widely used nanomaterials, especially in the field of drug delivery [1]. Various polymer carriers have been developed to encapsulate and deliver proteins, DNA, RNA, and small molecule drugs [2,3]. The polymer carrier shields the drugs from the environmental stress and can prevent drug degradation, reduce toxicity, and prolong the blood circulation of the drugs. Biodegradable polymers are especially attractive as the polymers can slowly degrade into smaller fragments, which can be either excreted or absorbed by the body [4,5]. This feature not only benefits the patients but also reduces the cost of post-care. Poly(lactic acid) (PLA) is a biodegradable polymer that has gained significant attention in recent years due to its potential to replace petroleum-based plastics [6,7]. The biodegradability of PLA is attributed to its chemical structure, which contains ester linkages that can be hydrolyzed when exposed to water and enzymes [8,9]. The biodegradability of PLA has made it a popular choice for various applications, including food packaging, biomedical implants, and drug delivery vehicles [10,11,12].

Carbohydrates are widely used as excipients in pharmaceutical formulations due to their ability to improve drug delivery, stability, and safety [13]. For glycomicelles, carbohydrates can increase micelle stability and decrease toxicity through the formation of a protective layer around the micelle surface. This layer helps to prevent the aggregation and precipitation of micelles, thereby improving their stability and solubility in physiological fluids [14]. In addition, the carbohydrate layer can act as a physical barrier, reducing the likelihood of toxic interactions [15,16]. Carbohydrates are often used as excipients in drug delivery systems to improve hydrophilicity and local concentration of the drug at the infected site [17,18,19]. Carbohydrates are biocompatible and suitable for drug delivery systems because carbohydrate functionalization on polymers can improve solubility, protect from enzyme degradation, and release drugs in a controlled manner [20,21,22]. Carbohydrates as the targeting agents can also enhance the activity of the drug by safely delivering it at the target site at a higher dose and lower systemic exposure [23,24].

Poly(ethylene glycol) (PEG) is a hydrophilic polymer that is often used as a linker in polymer micelles for drug delivery [25,26]. PEG has a number of properties that make it an effective linker in this context. The PEG linker in polymer micelles helps to form a hydrophilic corona around the hydrophobic micelle core, which can increase their stability and solubility in aqueous environments. The PEG corona can also act as a barrier to reduce the non-specific interactions of the micelle surface with serum proteins and cell membranes, which can improve the specificity and efficacy of drug delivery. PEG can provide steric stabilization to micelles, preventing aggregation and opsonization by the immune system, which can increase the circulation time of the micelles in the bloodstream and enhance their accumulation in target tissues. Additionally, PEGylation has been shown to improve the overall biocompatibility and reduce toxicity of the micelle system. Owing to these positive properties, PEG has been the preferred polymer in drug delivery systems for decades [27]. The hydrophilic nature of the PEG linker provides the drug delivery systems with enhanced enzymatic stability, reduced immunogenicity, and a longer circulation time by decreasing kidney clearance, all of which contribute to a more effective therapeutic outcome [28,29]. It has also been reported that the drug release is more efficient in the presence of PEG and that increasing the amount of PEG can lead to faster drug release rates [30,31,32].

Although PEG-PLA micelles are an established structure for drug delivery, many research efforts are focused on antitumor drugs and very few on other categories of drugs, such as the delivery of antibiotics [33,34]. Cai and coworkers prepared micelles with paclitaxel-loaded PEG-PLA nanoparticles decorated with tumor-targeting F3 peptide (F3-NP-PTX). The drug loading of NP-PTX was 1.24% and F3-NP-PTX was 1.05%, while the entrapment efficiency was 62.1% for NP-PTX and 52.5% for F3-NP-FTX. Moreover, the tumor inhibition rate of F3-NP-PTX was 57.3%, which was higher compared to both taxol and NP-PTX, proving to have a successful anti-tumor effect targeting diseased tissue [35]. Isoniazid (INH) and rifampicin are anti-tuberculosis drugs. Gupta group developed a duo drug delivery system by forming polymeric micelles using isoniazid conjugated PEG-PLA-di-block-copolymer (PEG-PLA-INH) to encapsulate rifampicin. The drug loading of rifampicin and INH in these polymer micelles were 16.7% and 23.1%, whereas encapsulation efficiency was 72.3% and 78.6%, respectively. Compared to the drugs alone, the drug-encapsulated micelles showed an approximately 8-fold reduction in minimum inhibitory concentration (MIC) against *Mycobacterium tuberculosis* [36]. Due to the poor water solubility and bioavailability of ciprofloxacin, a drug delivery system is often required to increase the antibacterial effect of the drug [37]. Farhangi et al. prepared nanomicelles to encapsulate ciprofloxacin employing fatty acid (stearic acid, palmitic acid, and linoleic acid) and grafted chitosan conjugates. The optimum formulation of drug-encapsulated micelles reported a drug loading of approximately 19% and the MICs were 2 and 4 times lower against *K. pneumoniae* and *P. aeruginosa*, respectively, compared to the drug alone [38].

In this work, a carbohydrate, including monosaccharide mannose (Man), disaccharide trehalose (Tre), and oligosaccharide maltoheptaose (G7), was conjugated to PLA through a PEG linker. The resulting Man-PEG-PLA, Tre-PEG-PLA, and G7-PEG-PLA are self-assembled into micelles that present the carbohydrate on the micelle surface. The ability of the glycopolymers to encapsulate antibiotics was tested using both a non-polar (rifampicin) and a polar (ciprofloxacin) antibiotic. Our results showed that despite the low drug loading efficiency, the glycopolymers were able to either maintain or increase the antibacterial activity of the antibiotics.

## 2. Results and Discussion

### 2.1. Synthesis of Glycopolymers

**Scheme 1:**
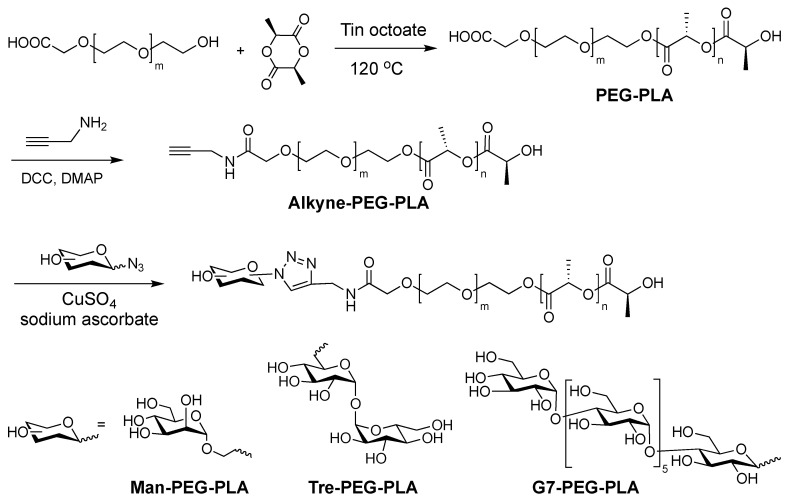
Synthesis of Man-PEG-PLA, Tre-PEG-PLA, and G7-PEG-PLA. DCC: *N*,*N’*-dicyclohexylcarbodiimide, DMAP: 4-dimethyaminopyridine.

Man-PEG-PLA, Tre-PEG-PLA, and G7-PEG-PLA were synthesized from an alkyne-functionalized PEG-PLA, alkyne-PEG-PLA, and the azide-derivatized sugar using the Cu(I)-catalyzed click reaction (Scheme 1). Azide-derivatized G7 (Scheme S1), mannose (Scheme S2), and trehalose (Scheme S3) were synthesized following previous procedures (see SI for synthesis and characterization) [39,40,41,42,43]. PEG-PLA was polymerized from *L*-lactide using a carboxy-PEG as the initiator and tin octoate as the catalyst (Scheme 1) [44]. Subsequent reaction with propargylamine gave alkyne-PEG-PLA, which reacted with the azido sugar to give Man-PEG-PLA, Tre-PEG-PLA, and G7-PEG-PLA after dialysis and lyophilization. For comparison, PLA glycopolymers without the PEG linker, G7-PLA, and Man-PLA, were prepared from alkyne-PLA and the azido sugar (Scheme S4).

**Figure 1:**
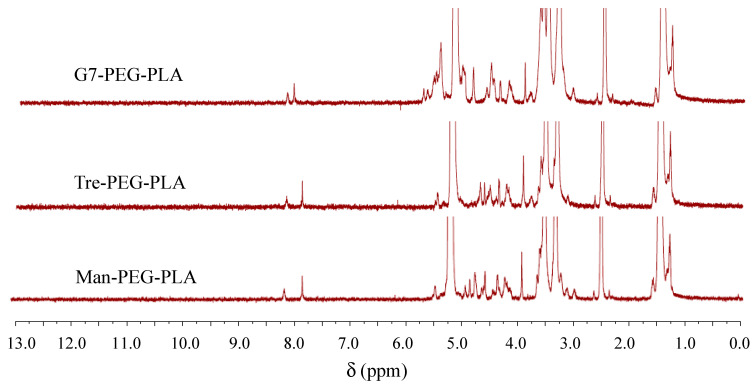
^1^H NMR spectra of G7-PEG-PLA, Tre-PEG-PLA, and Man-PEG-PLA in DMSO-*d*_6_. To improve visibility, the peaks corresponding to the polymers were amplified. The broad peaks at δ 8.08 (G7-PEG-PLA), 8.05 (Tre-PEG-PLA), and 8.16 ppm (Man-PEG-PLA) belong to the amide protons.

The successful synthesis of glycopolymers was confirmed by nuclear magnetic resonance (NMR) spectroscopy (Figure 1). The ^1^H NMR spectra contained the triazole protons at 7.96 (G7-PEG-PLA), 7.81 (Tre-PEG-PLA), and 7.87 (Man-PEG-PLA) [45,46]. The coupling yield of the click reaction, calculated from the ^1^H NMR spectra, remained relatively consistent, ranging from 40 to 50% (Table 1). All glycopolymers had similar molecular weights and low polydispersity.

### 2.2. Preparation of Glycopolymer Micelles

Since the carbohydrate content was low (Table 1), we tested whether the glycopolymers could self-assemble into micelles with carbohydrates extended at the surface of the micelle. The nanoprecipitation method was used to prepare micelles by dissolving glycopolymer in DMSO, followed by adding the solution into water dropwise under vigorous stirring [46]. While the micelles formed from PLA were large (243 nm, Entry 1, Table 2), the hydrodynamic diameter decreased to 25 nm for PEG-PLA (Entry 2, Table 2) and PDI was reduced from 0.32 to 0.15. The glycopolymer micelles had a similar size to the PEG-PLA at around 27–31 nm and PDI in the range of 0.20–0.28 (Entries 3–5, Table 2). The Zeta potential of PLA was −12 mV due to the carboxylate end group on the polymer. Conjugation of carbohydrates reduces the negative charge slightly to −10 to −11 mV.

**Figure 2:**
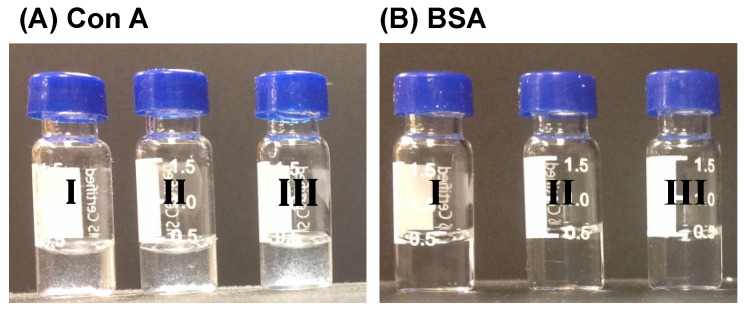
Photos of (I) Man-PEG-PLA, (II) Tre-PEG-PLA, (III) G7-PEG-PLA micelle solutions after treating with (**A**) Con A, or (**B**) BSA for 1 h. Micelles turned cloudy in the presence of Con A but remained clear in the presence of BSA.

To confirm that the carbohydrates are presented on the micelle surface, a lectin binding assay was conducted using concanavalin A (Con A), which binds mannoside and glucoside [47]. Bovine serum albumin (BSA) was used as the negative control. Con A has four binding sites and when interacting with multivalent entities such as glycopolymers, can form crosslinked aggregates [48,49]. This has been used widely as a tool to characterize glycopolymers and glyconanoparticles [50,51]. When Con A was added to the micelles prepared from Man-PEG-PLA, Tre-PEG-PLA, or G7-PEG-PLA, the clear solution turned turbid in minutes. After 1 h incubation, white aggregations settled to the bottom of the vials and were visible to the naked eye (Figure 2A). The micelle size increased from around 30 nm to 1.6–2.5 μm after 1 h and to 3.8–4.5 μm after 6 h of incubation with Con A, and PDI also increased (Table 3). None of the micelles aggregated in the presence of BSA, and the solution remained clear (Figure 2B). In addition, no change in the micelle size was observed, indicating the lack of interactions between glycomicelles and BSA (Table 3).

### 2.3. Antibiotic-Encapsulated Glycomicelles: Preparation and Antimicrobial Activities

#### 2.3.1. Encapsulation of Non-Polar Antibiotic and Antimicrobial Activity

Non-polar antibiotics were entrapped inside the glycomicelles by nanoprecipitation. Rifampicin was used as a model non-polar antibiotic. Although rifampicin is considered an amphiphilic antibiotic, the solubility in a non-polar solvent (349 mg/mL at 25 °C in chloroform) is much higher than in water (2.5 mg/mL at 25 °C), which allows easy encapsulation in hydrophobic polymer materials [52]. This was achieved by dissolving rifampicin and glycopolymer in 1:1 DMSO/THF, followed by adding the solution dropwise into the water while stirring. Rifampicin-encapsulated glycomicelles were obtained after dialysis to remove excess rifampicin and freeze drying.

After encapsulation with rifampicin, the sizes of the glycomicelles (Table 4) were similar to drug-free glycomicelles (Table 3), which was ~30 nm. All glycomicelles had similar a percent drug loading of 7–8% and entrapment efficiency of 20–24%. PEG-PLA had a slightly higher drug loading, but all were lower than PLA, which had percent drug loading of 14% and entrapment efficiency of 41% (Table 4). Micelles formed from PLA were also much larger, at ~300 nm. It was suggested that PEG could interfere with the polymer/drug complex and reduce the rifampicin solubility factor (solubility of rifampicin in the micelles/intrinsic solubility in water), resulting in a lower drug loading [53].

**Figure 3:**
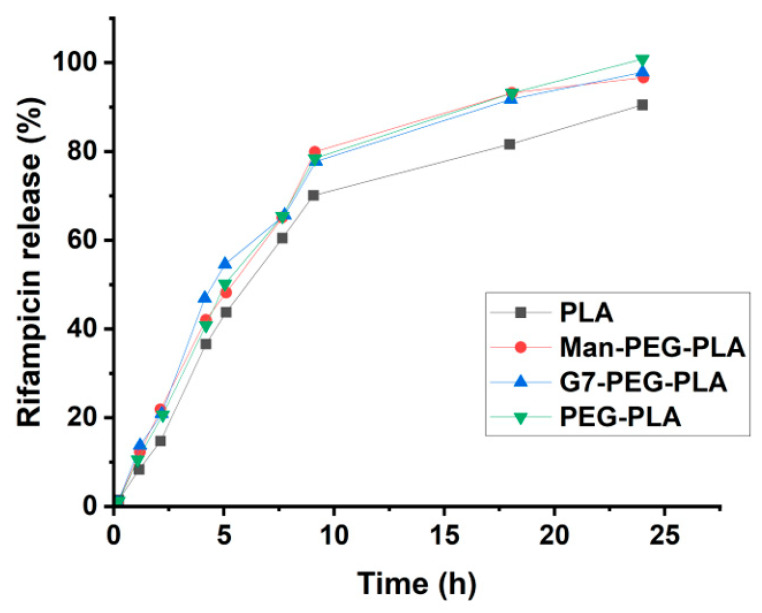
Percent rifampicin released from glycopolymer micelles over time. To assess the antibacterial activity of rifampicin-encapsulated micelles, the minimum inhibition concentration (MIC) against *E. coli* ORN 208, *S. epidermidis* 35984, and *M. smegmatis* mc^2^ 651 were determined (Table 5). The drug-free glycomicelles showed no toxicity and no inhibition was observed for the bacteria. Rifampicin-encapsulated micelles had similar MICs as rifampicin itself.

The drug release kinetics was assessed using the dialysis method. The rifampicin-encapsulated micelles were placed in a dialysis tube, and the concentration of released rifampicin was measured by taking aliquots of the aqueous solution at different time intervals. All glycomicelles showed a fast release in the first 10 h, followed by a slower release rate after 10 h, and over 90% was released from the micelles after 18 h. (Figure 3).

#### 2.3.2. Encapsulation of Polar Antibiotic and Antimicrobial Activity

Ciprofloxacin was used as the model polar antibiotic to test the encapsulation efficiency and antimicrobial activity. A double emulsion method (water/oil/water) was employed, whereby ciprofloxacin would be trapped in the inner water phase, and PLA would form a polymeric barrier (Figure S1) [54]. First, the water-in-oil emulsion was prepared by adding the aqueous solution (containing ciprofloxacin) into dichloromethane (containing the polymer) under high-power sonication. The water-in-oil emulsion was then added to a large amount of water under sonication to give water-in-oil-in-water micelles that should be stable under aqueous conditions. The size of the ciprofloxacin-encapsulated micelles was similar for both Man-PEG-PLA and G7-PEG-PLA (Table 6). The drug loading was 1.2 μg/mg and 2.5 μg/mg for Man-PEG-PLA and G7-PEG-PLA, corresponding to 0.12% and 0.25% of drug loading, respectively. This is significantly lower than in the case of rifampicin (8% and 7.2%, Table 4). The low loading efficiency has also been observed in other PLA systems and it was attributed to poor solubility of ciprofloxacin, causing it to diffuse out of the micelles during solvent evaporation and/or disruption of the water/oil/water emulsion by the strong sonication, causing the drug to leak [55].

Micelles formed from glycopolymers without the PEG linker, G7-PLA and Man-PLA (see Table S1 for characterization data), were 20–50% smaller, but the drug loading was 2–7 times higher (Entry 4 vs. 1, Entry 5 vs. 2, Table 6). These micelles were prepared from solid ciprofloxacin, not an aqueous solution, at a higher concentration, and a poly(vinyl alcohol) (PVA) stabilizer was included in the micelle preparation.

The antimicrobial activity of ciprofloxacin-encapsulated micelles was tested against five different strains of *E. coli*, including *E. coli* ORN208, which is a ciprofloxacin-resistant strain (Table 7) [56]. Indeed, the MIC of ciprofloxacin against *E. coli* ORN208 was 250 ng/mL, whereas the MICs of other strains were 8 or 16 ng/mL. Ciprofloxacin encapsulated in Man-PEG-PLA or G7-PEG-PLA showed 2–4 times better activity than the free ciprofloxacin. The PEG-PLA micelles were not toxic to the bacteria, and no inhibition was observed for PEG-PLA, Man-PEG-PLA, and G7-PEG-PLA micelles without encapsulated drugs. These polymers were synthesized by the click reaction using the copper catalyst and are the same polymers used to prepare antibiotic-encapsulated micelles, which were active. Based on these results, we believe that the residual copper concentration in the polymers is low and not a cause for concern. Without the PEG linker in the glycopolymer, the antimicrobial activity was worse. For example, the MICs of ciprofloxacin-encapsulated Man-PLA and G7-PLA were 4–6 times and 2–4 times worse than ciprofloxacin, respectively, whereas the MICs of ciprofloxacin encapsulated in PEG-PLA was either similar or two times better than ciprofloxacin (Table S2). It was suggested that PEG could cause the PLA core swell, and as a result, more water could enter into the hydrophobic core, leading to the creation of more porous structures that facilitate the release of the encapsulated drug [32].

## 3. Experimental Procedure

### 3.1. Synthesis of PEG-PLA

*L*-Lactide (1.5 g, 10 mmol), Sn(Oct)_2_ (10 mg), and carboxy-PEG (300 mg, 0.150 mmol, molecular weight 2000, Jenkem Technology, TX, USA) were added into a flame-dried flask. The reaction was purged with Ar, heated to 120 °C, and stirred for 3 h. After cooling to room temperature, the product was purified by dissolving in dichloromethane (DCM) and precipitating in hexanes for three times. The polymer was dissolved in DCM and washed with 1 M HCl and water to remove the trace metal catalyst. Finally, PEG-PLA was obtained as a white solid after removing the solvent (1.5 g, 83%). ^1^H NMR (500 MHz, CDCl_3_) δ 5.2 (–COC*H*(CH_3_)O–), 4.28 (–COC*H*(CH_3_)OH), 3.66 (–OC*H*_2_C*H*_2_O–), 1.57 (–COCH(C*H*_3_)O–); IR (ATR): 2876, 1746, 1452, 1381, 1361, 1267, 1183, 1127, 1081, 1046, 954, 863, 750 cm^−1^. M_n_: 8000, Ð: 1.30 (GPC, THF system).

### 3.2. Synthesis of Alkyne-PEG-PLA

PEG-PLA (800 mg) and propargylamine (11 mg, 0.20 mmol) were added into anhydrous DCM in the presence of DCC (60 mg, 0.29 mmol) and DMAP (26 mg, 0.21 mmol). The solution was stirred overnight, after which, the precipitation was filtered through a 0.45 μm PTFE membrane filter. The solution was then concentrated and poured into cold ether. The white powder was collected and washed with methanol for three times, followed by drying under vacuum to give the product as a white powder (722 mg, 92%). ^1^H NMR (500 MHz, CDCl_3_) δ 5.2 (–COC*H*(CH_3_)O–), 4.28 (–COC*H*(CH_3_)OH), 3.66 (–OC*H*_2_C*H*_2_O–), 2.26 (*H*C≡C–CH_2_–), 1.57 (–COCH(C*H*_3_)O–).

### 3.3. Synthesis of Carbohydrate-PEG-PLA

Carbohydrate-PEG-PLA was synthesized as follows. To 5 mL of DMSO, azido-sugar (0.09 mmol) was added together with alkyne-PEG-PLA (200 mg) under argon. CuSO_4_ (15 mg) and sodium ascorbate (40 mg) were added, and the reaction was stirred at room temperature for 2 days. Then, the mixture was poured into water and dialyzed against milli-Q water for two days. The final product was dried by lyophilization.

G7-PEG-PLA (160 mg, 82%): ^1^H NMR (500 MHz, DMSO-*d*_6_) δ 8.08 (triazole for MH), 5.2 (–COC*H*(CH_3_)O–), 3.2–5.6 (carbohydrate), 3.66 (–OC*H*_2_C*H*_2_O–), 1.57 (–COCH(C*H*_3_)O–). IR (ATR): 3392, 2877, 1747, 1452, 1381, 1362, 1267, 1184, 1127, 1081 (vs), 1042, 954, 863, 752 cm^−1^.

Tre-PEG-PLA (150 mg, 73%): ^1^H NMR (500 MHz, DMSO-*d*_6_) δ 8.15 (Tre), 5.2 (–COC*H*(CH_3_)O–), 3.2–5.6 (triazole for trehalose), 3.66 (–OC*H*_2_C*H*_2_O–), 1.57 (–COCH(C*H*_3_)O–). IR (ATR): 2877, 1747, 1452, 1381, 1362, 1267, 1184, 1127, 1081, 1044, 953, 863, 750 cm^−1^.

Man-PEG-PLA (153 mg, 75%): ^1^H NMR (500 MHz, DMSO-*d*_6_) δ 8.16 (triazole for Man), 5.2 (–COC*H*(CH_3_)O–), 3.2–5.5 (carbohydrate), 3.66 (–OC*H*_2_C*H*_2_O–), 1.57 (–COCH(C*H*_3_)O–). IR (ATR): 2877, 1747, 1452, 1381, 1361, 1267, 1183, 1127, 1082, 1046, 954, 863, 750 cm^−1^. Preparation of glycomicelles lectin binding

A solution of the glycopolymer (20 mg) dissolved in 1 mL of DMSO/THF (1:1 *v*/*v*) was added into 10 mL water dropwise under vortex at 500 rpm. Afterward, the solution was dialyzed in milli-Q water using a dialysis tube (MW cutoff: 3500) for 24 h. The micelles were then freeze-dried.

A solution of glycomicelles (100 μL, 0.1 mg/mL) in water was added to a solution of Con A in pH 7.4 PBS (100 μL, 0.1 mg/mL) containing 1.0 mM of MnCl_2_ and CaCl_2_, or BSA in pH 7.4 PBS (100 μL, 0.1 mg/mL). Aliquots were taken out at different time intervals and measured using DLS.

### 3.4. Preparation of Rifampicin-Encapsulated Micelles

Glycopolymer (20 mg) and 5 mg rifampicin were added into 1 mL of DMSO/THF (1:1 *v*/*v*), and the mixture was stirred until all solids were dissolved. The solution was added into 10 mL water dropwise under vortex at 500 rpm. Afterward, the solution was dialyzed in milli-Q water using a dialysis tube (MW cutoff: 3500) for 24 h. The micelles were then freeze-dried.

### 3.5. Determination of Drug Loading of Rifampicin-Encapsulated Micelles

Rifampicin-loaded micelles (2 mg) were added to 10 mL of DMSO under stirring. The absorbance at 580 nm was measured by UV-vis spectroscopy. The concentration of the drug loaded in the micelles was then calculated by comparing the measured absorbance to a calibration curve [57].

### 3.6. Determination of Drug Release of Rifampicin-Encapsulated Micelles

Freeze-dried and drug-loaded micelles (10 mg) were redispersed in 5 mL of pH 7.4 PBS buffer. It was then transferred into a dialysis tube (MW cutoff: 3500) and dialyzed in pH 7.4 PBS buffer at 37 °C under stirring at 150 rpm. Aliquots were taken out at different times and the absorbance was measured at 580 nm. The concentration of rifampicin was then calculated by comparing the measured absorbance to a calibration curve.

### 3.7. Determination of MIC of Rifampicin-Encapsulated Micelles

*E. coli* ORN 208, *S. epidermidis* 35*,*984, or *M. smegmatis* mc^2^651 were cultured in Luria-Bertani (LB) broth, tryptic soy broth, or Middlebrook 7H9 medium, respectively, to OD_600_ 0.3. The cultures were diluted to 1 × 10^6^ CFU/mL, and 100 μL was incubated with 100 μL of rifampicin-encapsulated glycopolymer micelles at various concentrations in a 96-well plate at 37 °C and 250 rpm for 18, 18 and 48 h, respectively. AlamarBlue (20 μL) was then added to each well, and the plate was incubated for 2 h. The fluorescence intensity was measured at 590 nm emission (560 nm excitation) using a spectrophotometer.

### 3.8. Preparation of Ciprofloxacin-Encapsulated Micelles

For PEG-PLA polymers, clinical ciprofloxacin (containing 10 mg/mL drug with lactic acid as the solubilizer and HCl for pH adjustment, purchased from Hospira, Inc., IL, USA) was used. An aqueous solution of ciprofloxacin (200 μL) was added into 2 mL of DCM containing 100 mg of PEG-PLA or the glycopolymer. The mixture was then sonicated (300 Watts, Sonics Vibra Cell VCX750, Sonics & Materials, Inc., Newtown, CT, USA) for 30 s. DCM was removed by stirring at 750 rpm for 30 min under air blowing. The resulting micelles were centrifuged and washed with water to remove the free ciprofloxacin, followed by lyophilization to give ciprofloxacin-encapsulated micelles.

For PLA polymers, ciprofloxacin powder (Sigma-Aldrich, 98% purity, cat. No. 17850-5G-F) was used. An aqueous solution of ciprofloxacin (600 μL, 30 mg/mL in 0.1% aqueous PVA) was added to 2 mL of DCM containing 100 mg of polymer. The mixture was then sonicated for 30 s. The water/DCM emulsion was then injected into an aqueous solution of PVA (20 mL, 0.1%, MW 30,000) and subjected to sonication for 2 min to form a water/oil/water double emulsion. DCM was removed by stirring at 750 rpm for 30 min under air blowing. The resulting micelles were centrifuged and washed with water to remove the free ciprofloxacin, followed by lyophilization to give ciprofloxacin-encapsulated micelles.

To measure the amount of encapsulated ciprofloxacin, 5–8 mg of ciprofloxacin-loaded micelles were dispersed in 4 mL of water, followed by sonication for 10 min. The suspension was centrifuged at 26,000 rpm for 30 min, and the supernatant was taken to measure the absorbance at 275 nm. The ciprofloxacin concentration was calculated by comparing it with a standard calibration curve prepared from 0, 0.5, 1, 2.5, 5, 10, and 20 μg/mL ciprofloxacin in Milli-Q water (Figure S2).

### 3.9. Determination of MIC of Ciprofloxacin-Encapsulated Micelles

Five *E. coli* strains were used in the experiments. JW 3994-2, JW 3996-1, JW3995-1, and JW 3993-1 were acquired from Yale Coli Genetic Stock Center (CGSC). *E. coli* ORN208 was a generous gift from Prof. Paul Orndorff, North Carolina State University. The bacteria were seeded on MH agar plates and incubated at 37 °C overnight. The bacteria were then transferred to MH broth and incubated under shaking at 250 rpm until OD_600_ reached 0.4–0.8. The bacteria were then isolated by centrifuging at 3000 rpm for 10 min and diluted in pH 7.4 PBS buffer followed by MH broth to 1 × 10^6^ CFU/mL. Ciprofloxacin or ciprofloxacin-loaded micelles of various concentrations were added into the bacteria and incubated at 37 °C under shaking (250 rpm) for 24 h. The bacteria suspension was diluted 10 times, and 10 μL of each was transferred into a 96-well plate, followed by 90 μL of PBS buffer. Serial dilutions were performed for a total of 6 dilutions, and 5 μL of each dilution was plated onto agar plates. Finally, the viability of the bacteria was obtained by counting colonies after overnight incubation.

## 4. Conclusions

In summary, we have synthesized glycopolymers by end-functionalizing biodegradable PLA with carbohydrates using a PEG linker. These glycopolymers are able to form micelles, with the carbohydrates presenting on the surface of the micelles, as confirmed by the lectin binding study. Both non-polar and polar antibiotics can be encapsulated in the glycomicelles. Rifampicin encapsulated micelles were smaller and had higher drug entrapment efficiency and loading compared to ciprofloxacin, possibly due to the more favorable interactions of the non-polar rifampicin with the hydrophobic PLA core than the zwitterionic ionic ciprofloxacin. The activity of ciprofloxacin- and rifampicin-encapsulated micelles against various bacteria strains was similar to or higher than that of the antibiotics alone. Without the PEG linker, the antibacterial activity was several times worse than the free antibiotics. Future work includes refining the structure of the biodegradable glycopolymers to improve their drug encapsulation and release properties, as well as leveraging the ability of carbohydrates to target specific cells to create targeted drug delivery systems.

## Figures and Tables

**Table 1:** Characterization of glycopolymers.

Glycopolymer	$\bar{M}$ _n_^**a**^	$\bar{M}$ _w_^**a**^	Ð	Coupling Yield ^b^	Carbohydrate Wt% ^c^
Man-PEG-PLA	20,000	22,000	1.11	40%	0.9%
Tre-PEG-PLA	20,100	23,400	1.16	50%	2.0%
G7-PEG-PLA	19,200	22,200	1.16	48%	6.1%

^a^ Measured by gel permeation chromatography (GPC) with DMF as the eluent and PMMA as the molecular weight standard. ^b^ Coupling yield = (reacted alkyne groups/all alkyne groups on the polymer) × 100%. It was calculated from the integrations of protons in the triazole versus that of the methyl group in the polylactide ((–CH(C*H_3_*)–, 1.0–1.5 ppm). ^c^ Carbohydrate wt% = (weight of conjugated carbohydrate/total weight of glycopolymer) × 100%.

**Table 2:** Characterization of glycopolymer micelles.

		Hydrodynamic Diameter (nm) ^a^	PDI ^a^	Zeta Potential (mV)
1	PLA	243.3 ± 0.6	0.32 ± 0.02	−12
2	PEG-PLA	25.1 ± 1.2	0.15 ± 0.02	−9.0
3	Man-PEG-PLA	31.4 ± 0.5	0.20 ± 0.04	−11
4	Tre-PEG-PLA	27.9 ± 0.7	0.22 ± 0.01	−10
5	G7-PEG-PLA	27.0 ± 0.1	0.28 ± 0.05	−10

^a^ Measured by dynamic light scattering (DLS). PDI: Polydispersity index.

**Table 3:** Hydrodynamic diameter and PDI of glycomicelles before and after treating with Con A or BSA, measured by DLS.

	Before		1 h after Treating with Con A		6 h after Treating with Con A		1 h after Treating with BSA	
	Diameter (nm)	PDI	Diameter (nm)	PDI	Diameter (nm)	PDI	Diameter (nm)	PDI
Man-PEG-PLA	31.4 ± 0.5	0.20 ± 0.04	2176 ± 47	0.60 ± 0.04	3839 ± 122	1.15 ± 0.07	37.3 ± 0.4	0.28 ± 0.02
Tre-PEG-PLA	27.9 ± 0.7	0.22 ± 0.01	2502 ± 41	0.30 ± 0.07	4476 ± 78	0.96 ± 0.09	31.7 ± 0.7	0.31 ± 0.02
G7-PEG-PLA	27.0 ±0.1	0.28 ± 0.05	1568 ± 22	0.50 ± 0.11	3878 ± 211	0.42 ± 0.03	27.9 ± 0.2	0.22 ± 0.03

**Table 4:** Characterizations of rifampicin-encapsulated micelles.

	Hydrodynamic Diameter ^a^ (nm)	PDI ^a^	Zeta Potential (mV) ^a^	Drug Loaded (μg/mg) ^b^	Percent Drug Loading ^c^	E. E. ^d^
Man-PEG-PLA	32.2 ± 0.4	0.18 ± 0.01	−25	80	8.0%	24%
Tre-PEG-PLA	28.9 ± 1.0	0.22 ± 0.01	−24	66	6.6%	20%
G7-PEG-PLA	27.1 ± 0.7	0.26 ± 0.05	−20	72	7.2%	22%
PEG-PLA	27.1 ± 0.5	0.17 ± 0.02	−21	92	9.2%	27%
PLA	300 ± 5.5	0.12 ± 0.01	−25	136	14%	41%

^a^ Determined by DLS. ^b^ Determined by disrupting the micelles and measuring the amount of rifampicin against a calibration. ^c^ Drug loading% = weight of drug loaded/weight of drug-encapsulated glycomicelles × 100%. ^d^ The entrapment efficiency or E.E.% = (actual drug loaded/initial drug added) × 100%.

**Table 5:** MICs of rifampicin-encapsulated micelles.

	*E. coli* ORN 208 (ng/mL)	*S. epidermidis* 35984 (ng/mL)	*M. smegmatis* mc^2^ 651 (μg/mL)
Man-PEG-PLA	32	8	25
Tre-PEG-PLA	ND	ND	25
G7-PEG-PLA	32	8	ND
PEG-PLA	32	8	25
Rifampicin	32	8	12.5

ND: not determined. Results for the rifampicin-encapsulated micelles were based on the weight of encapsulated rifampicin.

**Table 6:** Characterization of ciprofloxacin-encapsulated micelles.

		Hydrodynamic Diameter (nm) ^a^	PDI ^a^	Drug Loading (μg/mg) ^b^	Drug Loading% ^c^	E.E. ^d^
1	Man-PEG-PLA ^e^	416 ± 2	0.30 ± 0.01	1.2	0.12%	5.8%
2	G7-PEG-PLA ^e^	417 ± 4	0.46 ± 0.02	2.5	0.25%	6.3%
3	PEG-PLA ^e^	253 ± 8	0.73 ± 0.03	1.6	0.16%	7.5%
4	Man-PLA ^f^	318 ± 3	0.20 ± 0.02	7.7	0.8%	4.8%
5	G7-PLA ^f^	201 ± 4	0.30 ± 0.05	3.8	0.4%	2.1%
6	PLA ^f^	224 ± 2	0.35 ± 0.04	12	1.2%	6.6%

^a^ Determined by DLS. ^b^ Determined by disrupting the micelles and measuring the ciprofloxacin concentration. ^c^ Drug loading% = weight of drug-loaded/weight of micelles × 100%. ^d^ The entrapment efficiency or E.E.% = (actual drug loaded/initial drug added) × 100%. ^e^ Prepared from ciprofloxacin solution (Hospira, Inc. (Lake Forest, IL, USA), lot No. 35-216-DK). ^f^ Prepared from ciprofloxacin solid (Sigma-Aldrich (St. Louis, MO, USA), cat. No. 17850-5G-F).

**Table 7:** MIC (ng/mL) of ciprofloxacin-encapsulated PEG-PLA micelles against different *E. coli* strains.

	JW 3993-1	JW 3994-2	JW 3995-1	JW 3996-1	ORN 208
Ciprofloxacin	16	8	8	8	250
Man-PEG-PLA	8	2	4	4	125
G7-PEG-PLA	8	4	4	4	125

## Data Availability

The data presented in this study are available in the text and Supplementary Materials.

## Supplementary Materials

The following supporting information can be downloaded at https://www.mdpi.com/article/10.3390/molecules28104031/s1, Scheme S1: Synthesis of G7-azide, R=COCH3; Scheme S2: Man-azide (**10**); Scheme S3: Tre-azide (**16**); Scheme S4: G7-PLA and Man-PLA; Table S1: Characterization of G7-PLA and Man-PLA; Table 2: MIC of ciprofloxacin-encapsulated micelles; Figure S1: Preparation of antibiotic-encapsulated micelles by the double emulsion technique; Figure S2: Calibration curve of ciprofloxacin in water; Figure S3: ^1^H NMR spectra of compound **2**, **3** and **4** in CDCl_3_; Figure S4: ^1^H NMR and ^1^H-^1^H COSY spectra of G7-azide in D_2_O; Figure S5: FT-IR spectra of compound **2**, **3**, **4** and **5**; Figure S6: ^1^H NMR spectra of compound **7**, **8**, **9** in CDCl_3_ and **10** in D_2_O; Figure S7: FT-IR spectra of compound **7**, **8** and **10**; Figure S8: ^1^H NMR spectra of compound **13**, **14**, **15** in CDCl_3_ and **16** in D_2_O; Figure S9: FT-IR spectra of compound **13**, **14**, **15** and **16**; Figure S10: ^1^H NMR spectra of PEG, polymer **18** and **19** in CDCl_3_; Figure S11: FT-IR spectrum of PEG PLA; Figure S12: FT-IR spectrum of G7-PEG-PLA; Figure S13: FT-IR spectrum of Tre-PEG-PLA; Figure S14: FT-IR spectrum of Man-PEG-PLA; Figure S15: GPC traces of carbohydrate-PEG-PLA polymers (in DMF). Refs. [58,59,60,61,62] are cited in Supplementary Materials.
