# Supplementary material for: Synthesis of Glycopolymer Micelles for Antibiotic Delivery
Source: Molecules. 2023 May 11;28(10):4031. doi: 10.3390/molecules28104031 (PMC10224052; doi:10.3390/molecules28104031)
Supplement: Supplementary file 1 [file molecules-28-04031-s001.zip › molecules-2382865-supplementary.pdf]

1 Materials and Instruments

Iron (III) chloride ($FeCl_3$), sodium bicarbonate ($NaHCO_3$), sodium chloride (NaCl), sodium azide (NaN_3), sodium sulfate (Na_2SO_4), sodium methoxide (NaOMe, 25% in MeOH), calcium carbonate ($CaCO_3$), copper sulfate ($CuSO_4$), sodium ascorbate, sodium thiosulfate ($Na_2S_2O_3$), tin octoate ($Sn(Oct)_2$), ammonium chloride (NH_4Cl), acetic anhydride (Ac_2O), hydrogen bromide (HBr 33% in acetic acid), boron trifluoride diethyl etherate ($BF_3 \cdot Et_2O$), 2-bromoethanol, iodine, *p*-toluenesulfonyl chloride, benzaldehyde dimethyl acetal, *p*-toluenesulfonic acid, triethylamine, 4-dimethyaminopyridine (DMAP), carbon tetrachloride (CCl_4), *N*-bromo succinimide (NBS), mPEG-OH (Mw 2,000), propargylamine, *N*,*N'*-dicyclohexylcarbodiimide (DCC), D-mannose (Man), ciprofloxacin (Cip), hydrochloric acid (HCl), ethanol (EtOH), methanol (MeOH), Amberlite IRC-120H+ resin, tetrahydrofuran (THF), diethyl ether, acetone, hexane, copper(I) bromide (CuBr), phosphorus pentoxide, pentamethyldiethylenetriamine, Concanavalin A (Con A) and bovine serum albumin (BSA) were purchased from Sigma-Aldrich and used without further purification. Trehalose anhydrate, 2-bromopropionyl chloride, and β-cyclodextrin (CD) were purchased from TCI America and used as received. HOOC-PEG-OH (MW 2,000) was purchased from Jenkem Technology USA. H-Ser(benzyl)-OH was purchased from Chem Impex Int. Inc. Dichloromethane (DCM), dimethylformamide (DMF), ethyl acetate (EtOAc) and dimethyl sulfoxide (DMSO) were purified by distillation from CaH_2. Amberlite IRC-120 H+ resin was activated by washing with NaOH and HCl, followed by water. *L*-lactide was recrystallized from anhydrous EtOAc.

^1H NMR and ^1H-^1H COSY were performed on a Bruker 500 MHz spectrometer using deuterated solvent $CDCl_3$, D_2O or DMSO-d_6 (Cambridge Isotope Lab., Inc.). Polymers were characterized on Polymer Labs GPC-50 system with Agilent Plgel Guard column 50 x 7.5 mm/Agilent Plgel Mixed-D 300 x 7.5 mm column/Agilent Plgel Mixed-C 300 x 7.5 mm column and a Wyatt Technologies multi-angle light scattering (MALS) detector, running in DMF with 0.01 M LiCl at 1 mL/min (DMF system). FT-IR spectra were recorded on a Nicolet 6700 FT-IR spectrometer. DLS and zeta potential were measured by Delsa™ Nano HC (Beckman) equipped with size cell and flow cell. UV-vis spectra were recorded on a LAMBDA 45 UV/vis spectrophotometer (Perkin Elmer).

2 Synthesis of glycopolymer

2.1 Synthesis of G7-azide

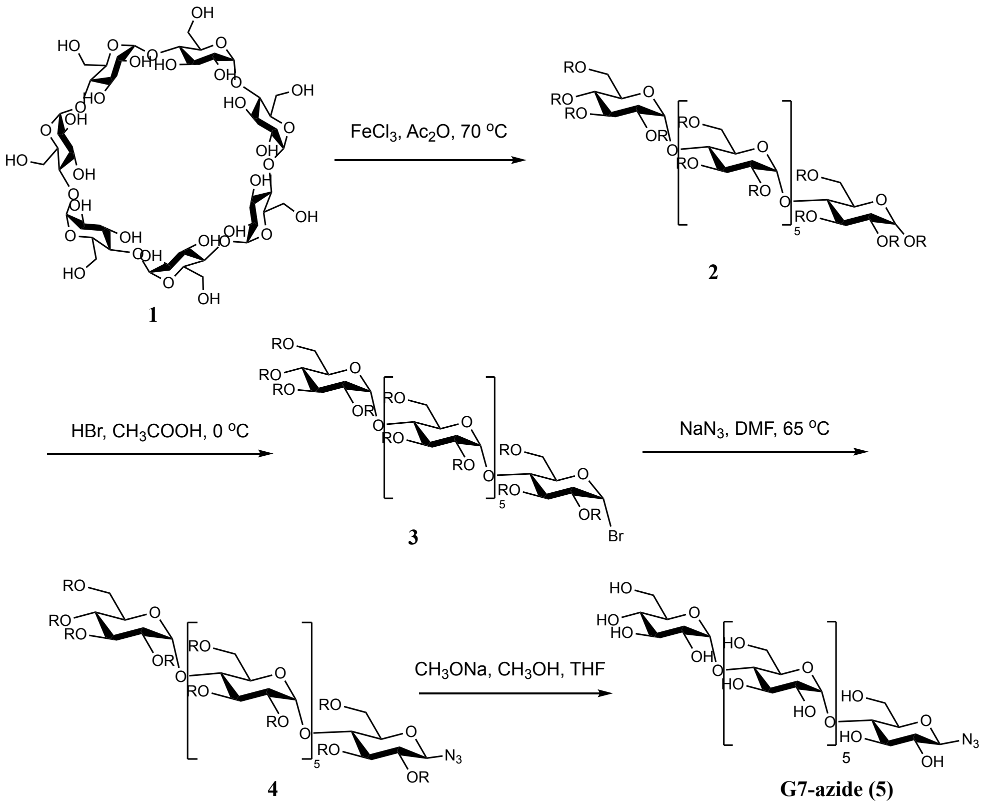

Scheme S1. Synthesis of G7-azide, R = $COCH_3$.

Synthesis of tricosa-*O*-acetyl-α-maltoheptaose (2)[1]

A solution of $FeCl_3$ (200 mg, 1.2 mmol) in Ac_2O (12.5 mL) was cooled in an ice bath, and β-cyclodextrin (4.0 g, 3.6 mmol) was added slowly. The mixture was stirred for 2.5 h at room temperature followed by 3.5 h at 70 °C. After cooling to room temperature, the red solution was poured into 500 mL of water. The precipitate was collected, washed with water and cold ethanol. After recrystallization from ethanol for 3 times, tricosa-*O*-acetyl-α-maltoheptaose (**2**) was obtained as a white solid (2.5 g, 33%). ^{1}H NMR ($CDCl_3$) δ 6.32 – 3.53 (m, 41H, H-1, H-2, H-3, H-4, H-5, H-6), 2.44 – 1.79 (m, 69H, OAc). IR: 2961, 1739, 1433, 1368, 1212, 1163, 1026, 943, 896, 762, 636 cm^{-1}.

Synthesis of docosa-*O*-acetyl-α-maltoheptaosyl bromide (3)[1]

A solution of **2** (1.85 g, 0.9 mmol) in anhydrous CH_2Cl_2 (5 mL) was cooled in an ice bath, and HBr in CH_3COOH (33% w/w, 3 mL) was added. The mixture was stirred at 0 °C for 3.5 h, after which, it was diluted to 50 mL with CH_2Cl_2, and washed with $NaHCO_3$ (10%, 20 mL), water and brine. After removing the solvent, compound **3** was obtained as white solid (1.8 g, 95%). ^{1}H NMR ($CDCl_3$) δ 6.49 (d, J = 3.6 Hz, 1H, H-1), 5.70 – 3.78 (m, 41H, H-1, H-2, H-3, H-4, H-5, H-6), 2.41 – 1.82 (m, 66H, OAc). IR (ATR): 2961, 1739, 1433, 1368, 1212, 1026, 945, 899, 763, 647 cm^{-1}.

Synthesis of docosa-*O*-acetyl-α-maltoheptaosylazide (4)[2]

Compound **3** (1.9 g, 0.9 mmol) was dissolved in 5 mL of dry DMF, and sodium azide (300 mg, 4.6 mmol) was added. The mixture was stirred at 65 °C for 3.5 hours. Water was added and a white precipitate was collected. The solid was then redissolved in CH_2Cl_2, washed with water followed by brine. The organic phase was dried over Na_2SO_4 and the solvent was evaporated to yield the product as a white solid (1.7 g, 87%). ^1H NMR ($CDCl_3$) δ 5.51 – 3.53 (m, 42H, H-1, H-2, H-3, H-4, H-5, H-6), 2.28 – 1.69 (m, 63H, OAc). IR (ATR): 2961, 2120, 1740, 1367, 1213, 1027, 895 cm^{-1}.

Synthesis of G7-azide (5)[2]

Compound **4** (2.8 g, 1.35 mmol) was added to 40 mL of methanol/THF (v/v 1:1) containing 1 mL of 25% CH_3ONa in methanol. After stirring at room temperature for 1 day, the precipitate was collected and washed with THF and ethanol to afford the product **5** as a white solid (1.5 g, 92%). ^1H NMR (500 MHz, D_2O) δ 5.41 (s, 6H, H-1), 4.70 (d, 1H, H-1_a) 4.03 – 3.26 (m, 41H, H-2, H-3, H-4, H-5, H-6). IR (ATR): 3311, 2926, 2120, 1364, 1243, 1146, 1077, 1016, 931, 848, 761 cm^{-1}.

2.2 Synthesis of Man-azide

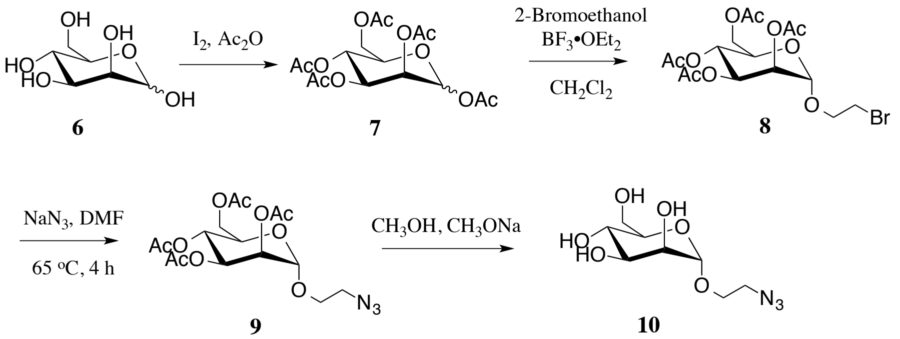

Scheme S2. Synthesis of Man-azide (**10**).

Synthesis of penta-*O*-acetyl-D-mannopyranose (7)[3]

Iodine (50 mg, 0.2 mmol) was dissolved in Ac_2O (20 mL) in a water bath (18 °C), and D-mannose (4 g, 22.3 mmol) was added slowly to prevent the temperature from rising above 40 °C, until all sugar was completely dissolved. After acetylation was completed, the solution was washed with cold saturated $Na_2S_2O_3$ to remove iodine. To neutralize the byproduct acetic acid, 10% $NaHCO_3$ solution was added until no CO_2 was generated. Then 50 mL of CH_2Cl_2 was added, and the aqueous layer was decanted. The organic layer was washed with water and dried over anhydrous Na_2SO_4. The filtrate was collected, and the solvent was removed under vacuum to give penta-*O*-acetyl-D-mannopyranose **7** as a white solid (7.5 g, 88%). ^1H NMR (500 MHz, $CDCl_3$) δ 6.10 – 6.04 (m, 2H), 5.85 (s, 1H), 5.47 (s, 1H), 5.37 – 5.21 (m, 15H), 5.12 (dd, J = 9.9, 3.2 Hz, 1H), 4.28 (td, J = 12.8, 5.1 Hz, 3H), 4.16 – 4.00 (m, 5H), 3.79 (dq, J = 7.5, 3.0, 2.5 Hz, 1H), 2.23 – 1.95 (m, 45H).

Synthesis of 2'-bromoethyl 2,3,4,6-tetra-*O*-acetyl-α-D-mannopyranoside (8)[3]

2-Bromoethanol (0.74 mL, 10.2 mmol) and $BF_3 \cdot Et_2O$ (3.2 mL, 25.6 mmol) were added to a 100-

mL sealed flask containing 20 mL CH_2Cl_2 and compound **7** (2.0 g, 5.1 mmol). After the reaction was completed, 100 mL of CH_2Cl_2 was added to dilute the mixture and a saturated solution of $NaHCO_3$ was used to neutralize BF_3. Then, the organic layer was dried over Na_2SO_4 and concentrated using a Rotavap. Purification was done by chromatography on silica gel (1:1 hexanes/EtOAc). A white powder was obtained after evaporation of the solvent (1.7 g, 75%). ^1H NMR (500 MHz, $CDCl_3$) δ 5.31 (m, 3H, H-2, H-3, H-4), 4.88 (s, 1H, H-1), 4.28 (dd, J = 12.4, 5.9 Hz, 1H, H-6_a), 4.14 (d, J = 10.3 Hz, 2H, H-5, H-6_b), 3.98 (dd, J = 11.5, 5.7 Hz, 1H, H-7_a), 3.90 (m, 1H, H-7_b), 3.52 (m, 2H, H-8), 2.22 – 1.97 (m, 15H, OAc). IR (ATR): 2921, 2109, 1733, 1436, 1367, 1225, 1136, 1084, 1050, 978, 972, 934, 908, 878, 851, 830, 767, 686 cm^{-1}.

Synthesis of 2'-azidoethyl 2,3,4,6-tetra-*O*-acetyl-α-D-mannopyranoside (9)[3]

To sodium azide (2.0 g, 30.5 mmol) and **8** (6.9 g, 15.1 mmol) was added 50 mL of DMF. The mixture was heated at 65 °C for 4 h and then poured into saturated NaCl. The raw product was extracted 3 times with a total of 150 mL CH_2Cl_2, and the organic phase was dried over Na_2SO_4. After removing the solvent, the residual was purified by flash chromatography (4:1 hexanes/EtOAc) to give the product as a white solid (5.0 g, 80%). ^1H NMR (500 MHz, $CDCl_3$) δ 5.33 (m, 3H, H-2, H-3, H-4), 4.87 (s, 1H, H-1), 4.29 (dd, J = 12.2, 5.3 Hz, 1H, H-6_a), 4.13 (d, J = 12.3 Hz, 1H, H-6_b), 4.09 – 4.00 (m, 1H, H-5), 3.95 – 3.81 (m, 1H, H-7_a), 3.73 – 3.60 (m, 1H, H-7_b), 3.47 (ddd, J = 13.2, 6.2, 3.6 Hz, 2H, H-8), 2.34 – 1.83 (m, 12H, OAc). IR (ATR): 2921, 2109, 1742, 1448, 1367, 1330, 1228 , 1135, 1083, 1024, 988, 972, 877, 836, 807, 676 cm^{-1}.

Synthesis of Man-azide (10)[3]

Compound **9** (4.0 g, 9.6 mmol) was dissolved in 50 mL of methanol, and NaOMe (518 mg, 9.6 mmol) was added. The mixture was stirred for additional 1 h and was neutralized with activated Amberlite IRC-120 H+ resin. After removing the solvent, the mixture was purification by flash chromatography (9:1 CH_2Cl_2/MeOH), yielding the product as a white powder (1.8 g, 76%). ^1H NMR (500 MHz, D_2O) δ 4.92 (1H, H-1), 4.06 – 3.81 (m, 4H, H-2, H-3, H-6_a, -OCH_2CH$_2$N$_3$), 3.74 (m, 4H, H-4, H-5, H-6_b, -OCH_2CH$_2$N$_3$), 3.52 (m, -OCH$_2$CH_2N$_3$). IR (ATR): 3286, 2921, 2109, 1449, 1367, 1278, 1333, 1057, 1024, 971, 919, 876, 836, 807 cm^{-1}.

2.3 Synthesis of Tre-azide (16)

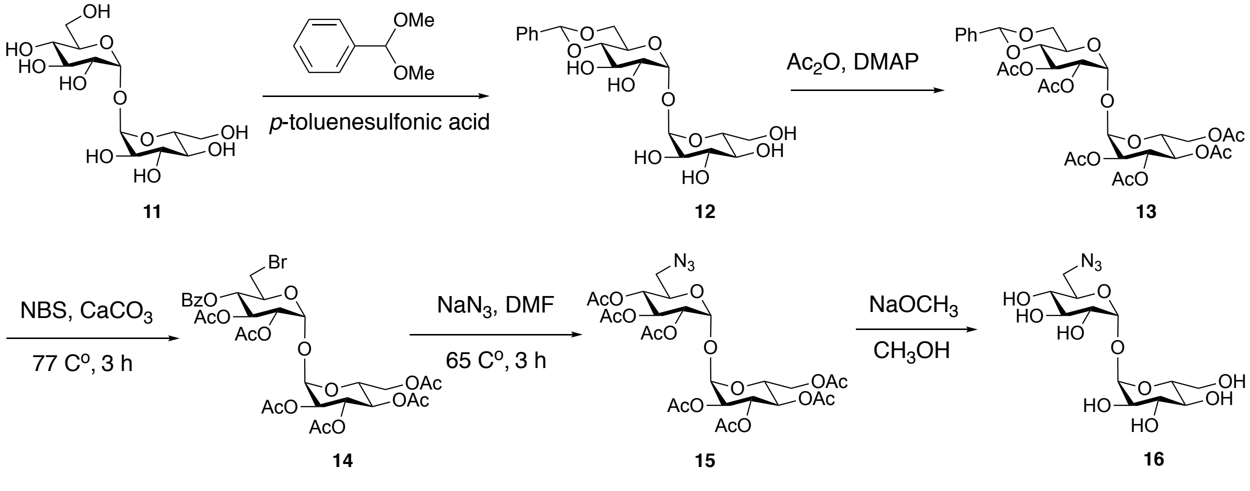

Scheme S3. Synthesis of Tre-azide (**16**).

Synthesis of 4,6-benzylidene-α,α-D-trehalose (12)[4, 5]

Benzaldehyde dimethyl acetal (1.1 g, 7.0 mmol), trehalose (2 g, 5.8 mmol) and *p*-toluenesulfonic acid (100 mg, 0.58 mmol) were added into 20 mL of DMF. The mixture was stirred for 12 hours at room temperature and further heated up to 30-40 °C for 3 hours. TLC was constantly used to check the reaction. After the solution became transparent, the crude solution was used in the next step directly without further purification.

Synthesis of 2,2',3,3',6'-penta-*O*-acetyl-4,6-benzylidene-α,α-D-trehalose (13)[4, 5]

Triethylamine (14 g, 0.14 mol), Ac_2O (7 g, 0.7 mol) and DMAP (122 mg, 1 mmol) were added into the above crude mixture. After stirring overnight, the reaction was transferred into a separatory funnel and a large amount of saturated NaCl was added. The crude product was extracted with ethyl acetate for three times. The organic phase was combined, washed with water/brine and concentrated under vacuum. After flash column chromatography (2:3 hexanes/ethyl acetate), compound **13** was obtained as a white powder (1.2 g, 30%). ^{1}H NMR (500 MHz, $CDCl_3$) δ 7.49 – 7.39 (m, 2H, Ph), 7.39 – 7.30 (m, 3H, Ph), 5.61 (t, *J* = 9.8 Hz, 1H, H-3_a), 5.56 – 5.46 (m, 2H, H-3_b, Ph-C*H*-(OCH_2)(OCH)-), 5.37 (d, *J* = 3.7 Hz, 1H, H-1_a), 5.27 (d, *J* = 3.7 Hz, 1H, H-1_b), 5.10 – 4.94 (m, 3H, H-2_a, H-2_b, H-4_a), 4.25 (dd, *J* = 12.1, 5.5 Hz, 1H, H-6_{a1}), 4.16 (dt, *J* = 15.9, 7.9 Hz, 1H, H-6_{a2}), 4.10 (dd, *J* = 10.2, 5.3 Hz, 1H, H-5_a), 4.06 – 3.93 (m, 2H, H-5_b, H-6_{b1}), 3.75 (t, *J* = 10.4 Hz, 1H, H-6_{b2}), 3.69 (t, *J* = 9.6 Hz, 1H, H-4_b), 2.22 – 1.96 (m, 18H, OAc). IR (ATR): 2980, 1741, 1374, 1213 , 1133 (m), 1098, 1020, 980, 956, 906, 803, 767, 710, 652, 604 cm^{-1}.

Synthesis of 2,2',3,3',4',6'-penta-*O*-acetyl-6-azido-4-benzoyl-6-deoxy-α,α-D-trehalose (14)[4, 5]

Compound **13** (1.0 g, 1.5 mmol) was added into 50 mL of CCl_4 containing NBS (285 mg, 1.6 mmol) and $CaCO_3$ (160 mg, 1.6 mmol). The mixture was refluxed at 77 °C for 3 hours. After cooling to room temperature, the solution was washed with 10% $NaHCO_3$ and water. The organic phase was dried with Na_2SO_4 and concentrated in vacuum. Purification was done by column chromatography (2:3 hexane/ethyl acetate) to give the product as a white powder (1.0 g, 85%). ^{1}H NMR (500 MHz, $CDCl_3$) δ 8.03 (d, *J* = 7.3 Hz, 2H, Ph), 7.62 (t, *J* = 7.3 Hz, 1H, Ph), 7.49 (t, *J* = 7.7 Hz, 2H, Ph), 5.71 (t, *J* = 9.8 Hz, 1H, H-3_a), 5.54 (t, *J* = 9.7 Hz, 1H, H-3_b), 5.39 (d, *J* = 11.8, 2H, H1_a, H1_b), 5.24 – 5.16 (m, 2H, H-2_a, H-4_b), 5.14 – 5.03 (m, 2H, H-2_b, H-4_a), 4.23 (m, 2H, H-5a, H6), 4.16 – 3.97 (m, 2H), 3.49 – 3.25 (m, 2H), 2.34 – 1.75 (m, 18H). IR (ATR): 2960, 1744, 1430, 1367, 1211, 1160, 1062, 1022, 980, 958, 900, 803, 712, 599 cm^{-1}.

Synthesis of 2,2',3,3',4',6'-penta-*O*-acetyl-6-azido-4-benzoyl-6-deoxy-α,α-D-trehalose (15)[4, 5]

A solution of compound **14** (1.0 g, 1.2 mmol) and NaN_3 (150 mg, 3 mmol) in DMF (10 mL) was heated under Ar at 65 °C for 3 h. After cooling to room temperature, the insoluble material was filtered off. 100 mL of CH_2Cl_2 was added into the filtrate and the mixture was washed with water followed by brine. The organic layer was dried over Na_2SO_4. The filtered solution was concentrated under reduced pressure, yielding compound **15** (0.7 g, 82%). ^{1}H NMR (500 MHz, $CDCl_3$) ^{1}H NMR (500 MHz, $CDCl_3$) δ 8.04 (d, *J* = 8.2 Hz, 2H), 7.63 (t, *J* = 7.3 Hz, 1H), 7.49 (t, *J* = 7.6 Hz, 2H), 5.71 (t, *J* = 9.8 Hz, 1H), 5.57 (t, *J* = 9.8 Hz, 1H), 5.39 (d, *J* = 17.4, 2H), 5.25 (t, *J* = 9.8 Hz, 1H), 5.19 – 5.02 (m, 3H), 4.28 (dd, *J* = 12.1, 5.6 Hz, 1H), 4.22 – 4.09 (m, 2H), 4.05 (d, *J* = 12.1 Hz, 1H), 3.46 (dd, *J* = 13.4, 7.6 Hz, 1H), 3.23 (dd, *J* = 13.1, 1.7 Hz, 1H), 2.34 – 1.75 (m, 18H). IR (ATR): 2060, 2104, 1746, 1367, 1211, 1135, 1065, 1018, 982, 897, 802, 754, 712, 599 cm^{-1}.

Synthesis of Tre-azide (16)[4, 5]

Compound **15** (1.0 g, 1.3 mmol) and NaOMe (15 mg, 0.27 mmol) were added to methanol (20 mL). After 1 hour stirring, the mixture was neutralized with Amberlite IRC-120 H+ resin and filtered. After removing the solvent, the crude was purified by column chromatography (1:4:4 water/isopropanol/ethyl acetate) to give **20** as a white powder (239 mg, 50%). ^{1}H NMR (500 MHz, $CDCl_3$) δ 5.16 (m, 2H), 3.93 (m, 1H), 3.80 (m, 4H), 3.76 – 3.69 (m, 1H), 3.63 (m, 3H), 3.57 – 3.46 (m, 1H), 3.42 (m, 2H). IR (ATR): 3287, 2928, 2101, 1651, 1435, 1282, 1146, 1101, 1073, 984, 941, 840, 803 cm^{-1}.

2.4 Synthesis of G7-PLA and Man-PLA

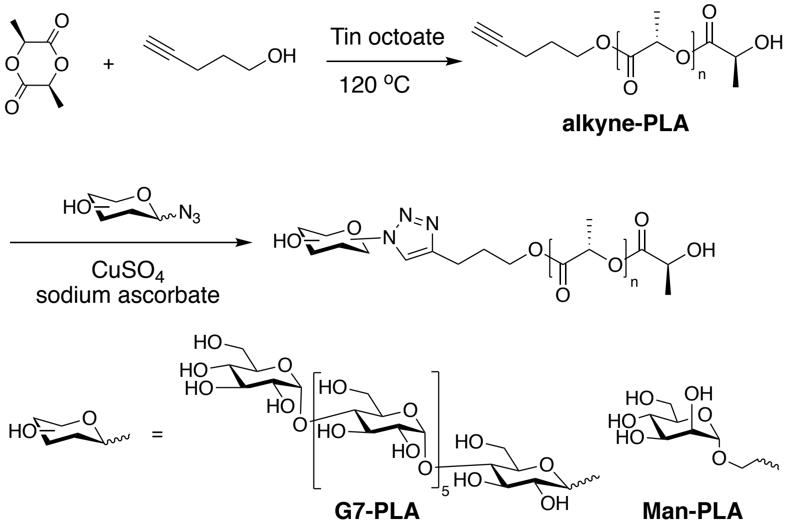

Scheme S4. Synthesis of G7-PLA and Man-PLA.

Synthesis of alkyne-PLA

To a flame-dried flask was added 4 g of *L*-lactide, 20 mg of $Sn(Oct)_2$ and 40 mg of 4-pentyn-1-ol. The solution was purged with Ar and heated to 120 °C for 3 hours. After cooling to room temperature, the product was purified by dissolving in CH_2Cl_2 and precipitating from hexanes three times to give alkyne-PLA after drying (3.2 g, 81%). ^{1}H NMR (500 MHz, $CDCl_3$) δ 5.2 (-COC*H*(CH_3)O-), 4.3 (-COC*H*(CH_3)OH), 4.25 (HC≡CCH_2CH_2C*H*$_2$OCO-), 2.7 (-COCH(CH_3)O*H*), 2.25 (HC≡CCH_2C*H*$_2$$CH_2$OCO-), 2.0 (*H*C≡C$CH_2CH_2CH_2$OCO-), 1.85 (HC≡CC*H*$_2$$CH_2CH_2$O CO-), 3.66 (-OC*H*$_2$C*H*$_2$O-), 1.57 (-COCH(C*H*$_3$)O-); FT-IR 2956 (w), 1746 (vs), 1452 (m), 1380 (m), 1266 (m), 1182 (s), 1128 (m), 1080 (vs), 1046 (m), 955 (w), 864 (m), 750 (w) cm^{-1}; M_n: 7000, Đ: 1.48 (by GPC in DMF).

Synthesis of G7-PLA

To 5 mL of DMSO was added G7-azide (200 mg, 0.2 mmol) and alkyne-PLA (800 mg, 0.1 mmol). The solution was bubbled with Ar for 1 h. $CuSO_4$ (15 mg) and sodium ascorbate (40 mg) were added, and the mixture was bubbled with Ar for another hour. The reaction was continuously stirred for 2 days, after which, the mixture was poured into water and then dialyzed against milli-Q water for two days. G7-PLA was obtained as a white powder after lyophilization (600 mg, 76%). ^{1}H NMR (500 MHz, DMSO-d_6) δ 8.1 (triazole), 5.2 (-COC*H*(CH_3)O-), 3.2-5.6

(carbohydrate), 1.57 (-COCH(CH_3)O-); FT-IR (cm^{-1}) 3358 (br), 2994 (w), 1748 (vs), 1452 (m), 1381 (m), 1267 (m), 1184 (s), 1128 (m), 1083 (vs), 1043 (m), 865 (m), 752 (w) cm^{-1}; M_n: 8100, Đ: 1.35 (by GPC in DMF).

Synthesis of Man-PLA

Man-PLA was synthesized from Man-azide and alkyne-PLA following the same procedure as above. The product was obtained as a white powder (530 mg, 66%). ^{1}H NMR (500 MHz, DMSO-d_6) δ 7.6 (triazole), 5.2 (-COC*H*(CH_3)O-), 3.2-5.5 (carbohydrate), 1.57 (-COCH(CH_3)O-); FT-IR 2995 (w), 1746 (vs), 1451 (m), 1381 (m), 1267 (m), 1183 (s), 1128 (m), 1082 (vs), 1044 (m), 864 (m), 750 (w) cm^{-1}; M_n 7200, Đ: 1.36 (by GPC in DMF).

Table S1. Characterization of G7-PLA and Man-PLA

Glycopolymer	$\overline{M}_n$ [a]	$\overline{M}_w$ [a]	PDI	Coupling yield[b]	Carbohydrate wt%[c]
G7-PLA	21,900	27,800	1.27	13%	38%
Man-PLA	34,100	60,000	1.76	90%	29%

[a] Measured by GPC with DMF as the eluent and PMMA as the standard.

[b] Coupling yield = (reacted alkyne groups / all alkyne groups on the polymer) × 100%

[c] Carbohydrate wt% = (weight of conjugated carbohydrate / total glycopolymer weight) × 100%

Table S2. MIC (ng/mL) of ciprofloxacin-encapsulated micelles

	E. coli Bort	*S. epidermidis* 35984	*K. pneumoniae* xx
Ciprofloxacin	19	310	160
Man-PLA[a]	110	1,250	630
G7-PLA[a]	78	625	310
PEG-PLA[a]	19	160	70

[a] Calculated from the weight of ciprofloxacin encapsulated in the micelles

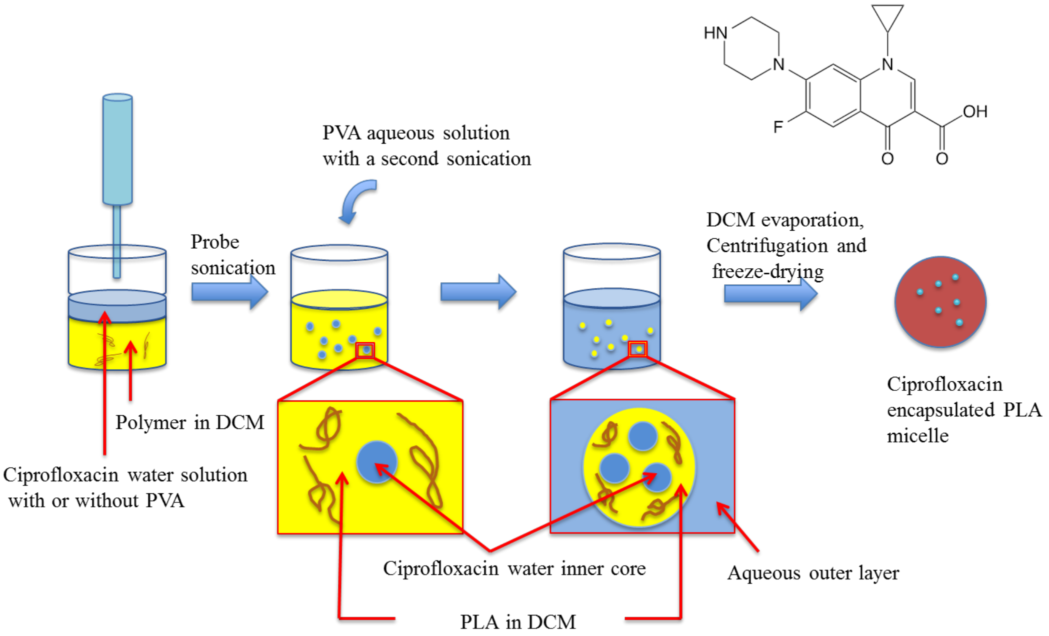

Figure S1. Preparation of antibiotic-encapsulated micelles by the double emulsion technique. Cipro is used for illustration. PVA: poly(vinyl alcohol), DCM: dichloromethane.

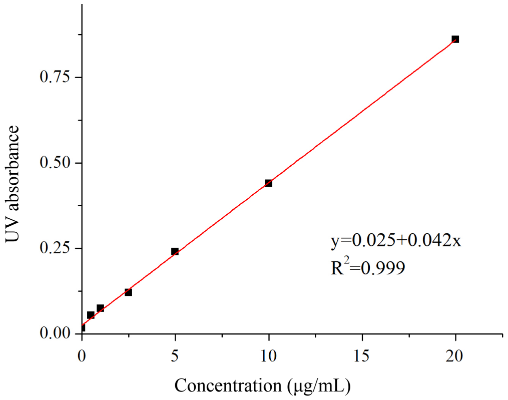

Figure S2. Calibration curve of ciprofloxacin in water. Absorbance was measured at 275 nm.

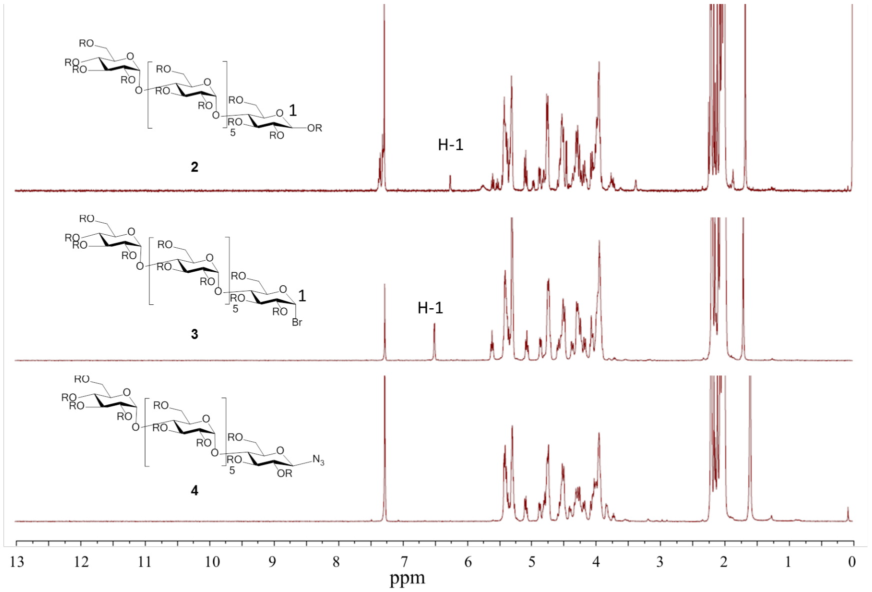

Figure S3. ^{1}H NMR spectra of compound **2**, **3** and **4** in $CDCl_3$

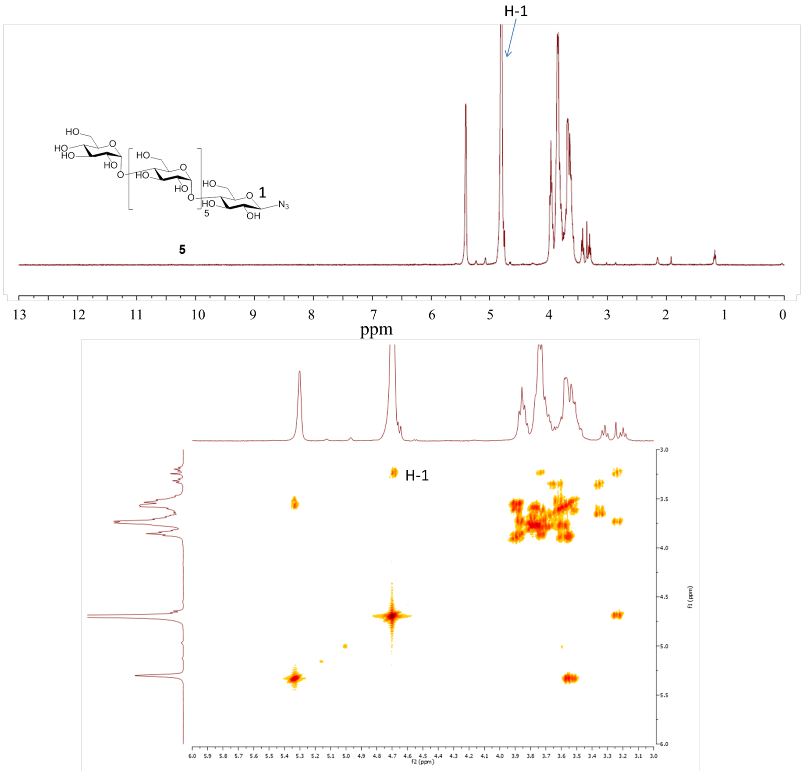

Figure S4. ^{1}H NMR and ^{1}H-^{1}H COSY spectra of G7-azide in D_2O

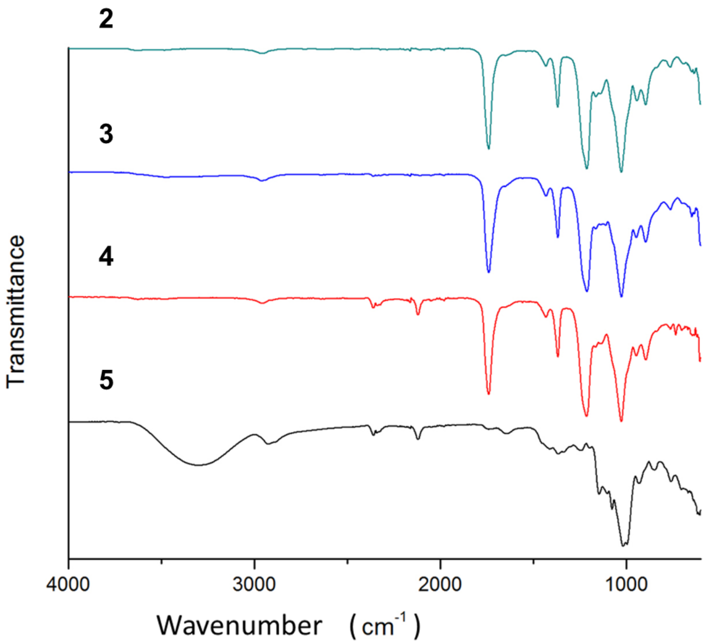

Figure S5. FT-IR spectra of compound **2**, **3**, **4** and **5**

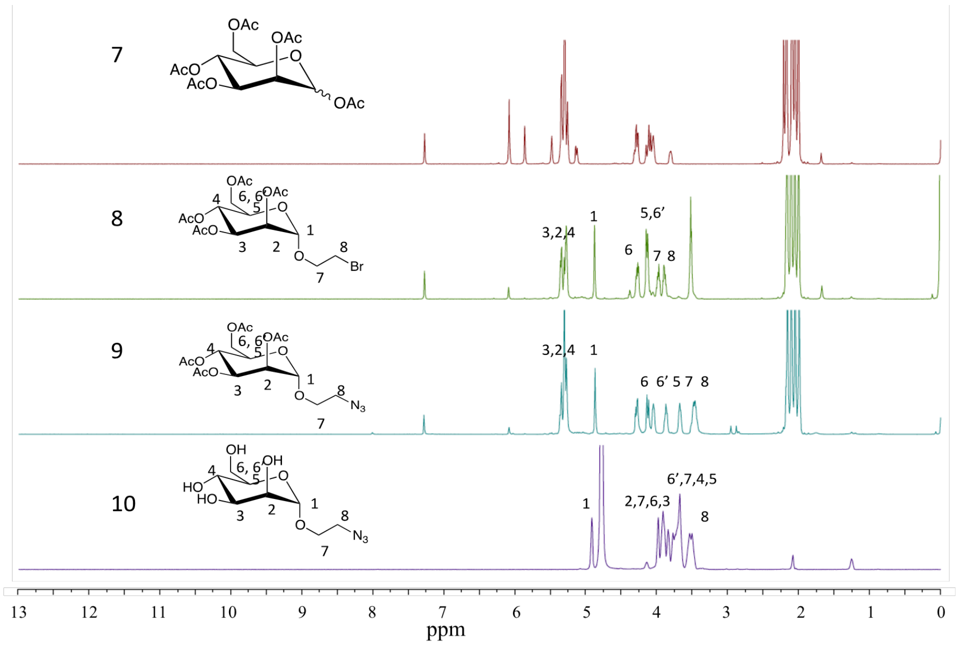

Figure S6. ^{1}H NMR spectra of compound **7**, **8**, **9** in $CDCl_3$ and **10** in D_2O

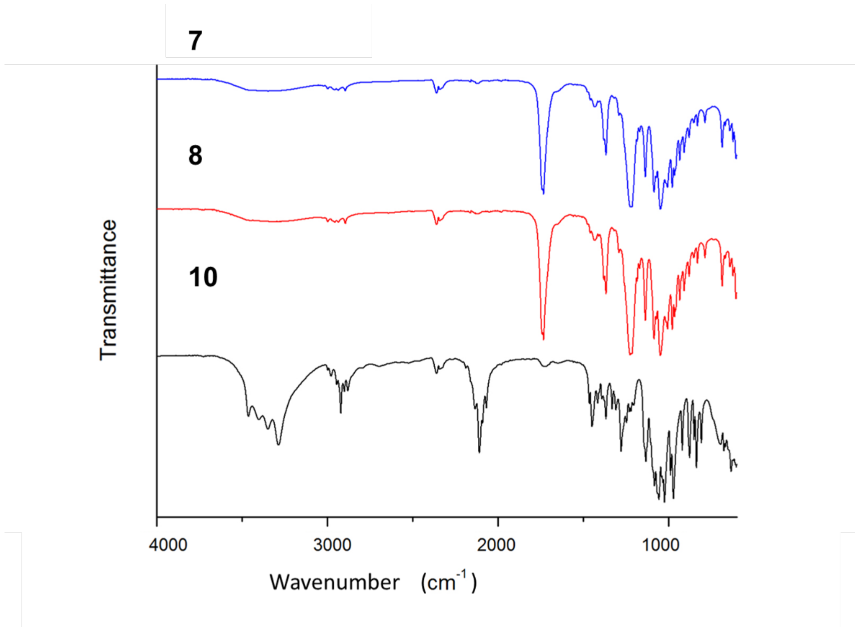

Figure S7. FT-IR spectra of compound **7**, **8** and **10**

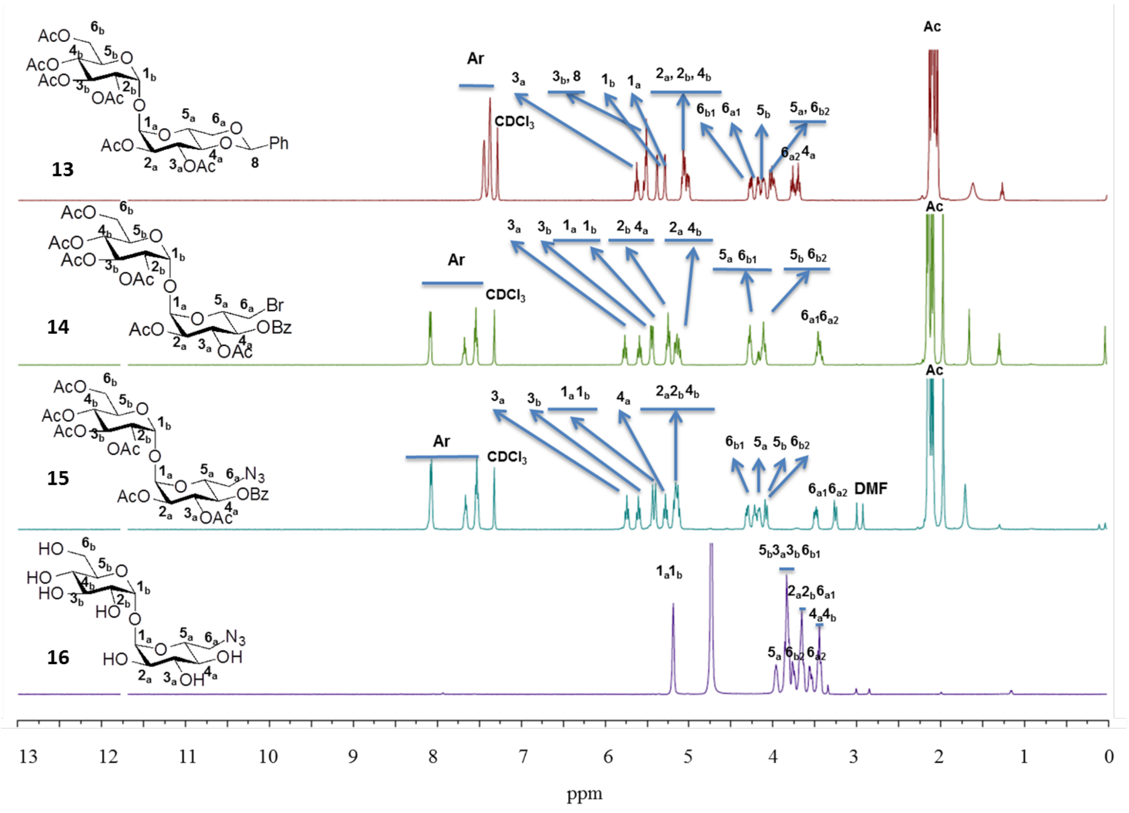

Figure S8. ^{1}H NMR spectra of compound **13**, **14**, **15** in $CDCl_3$ and **16** in D_2O

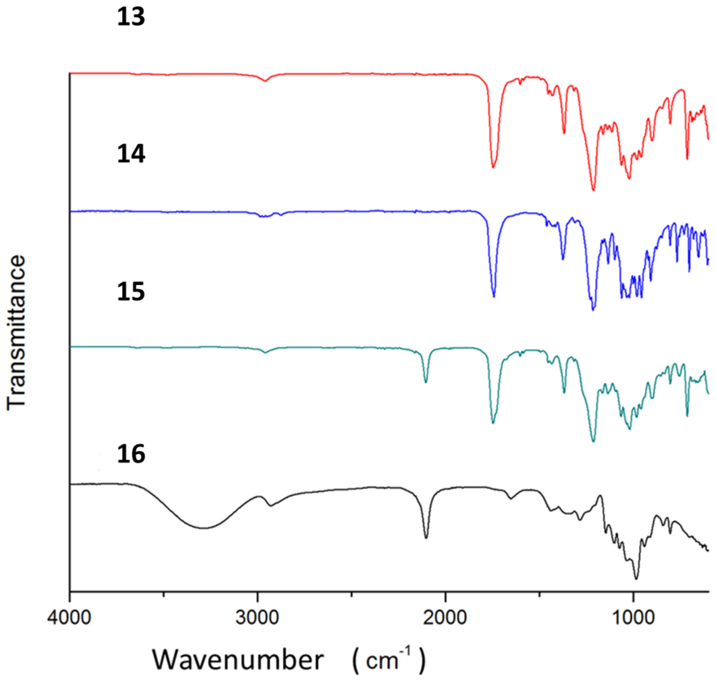

Figure S9. FT-IR spectra of compound **13**, **14**, **15** and **16**

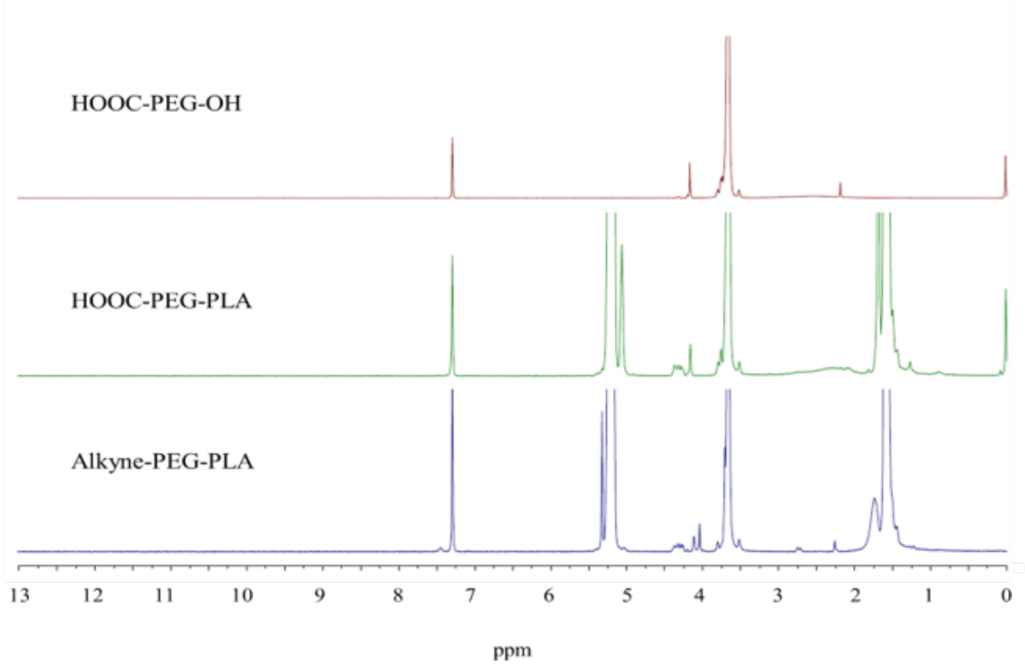

Figure S10. ^{1}H NMR spectra of PEG, polymer **18** and **19** in $CDCl_3$

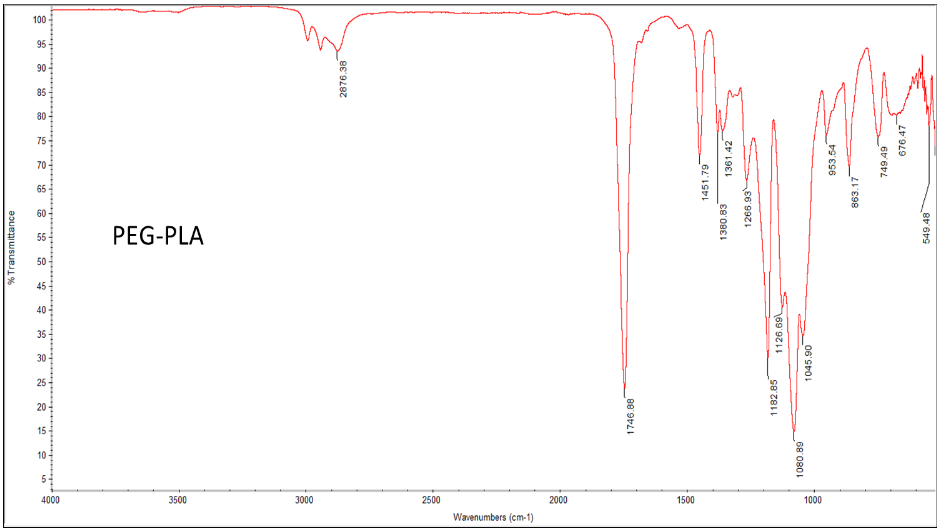

Figure S11. FT-IR spectrum of PEG PLA.

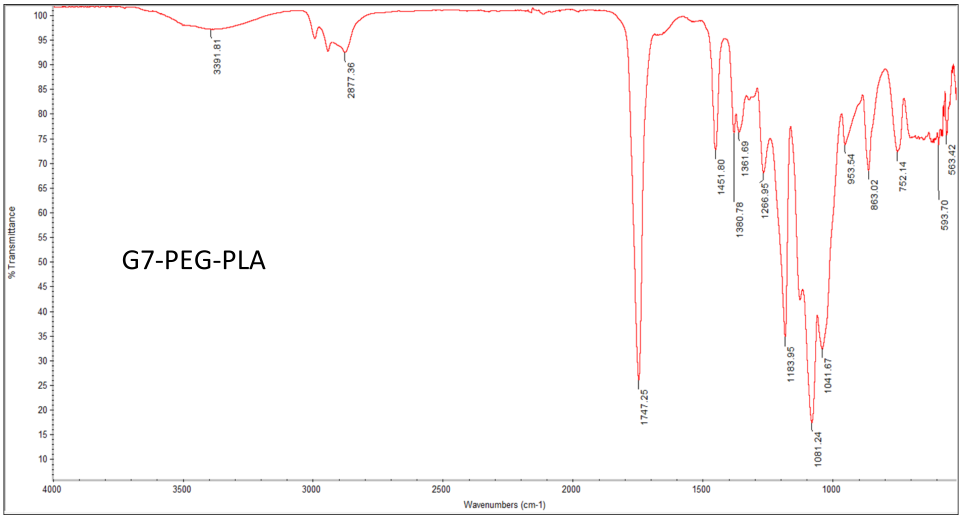

Figure S12. FT-IR spectrum of G7-PEG-PLA

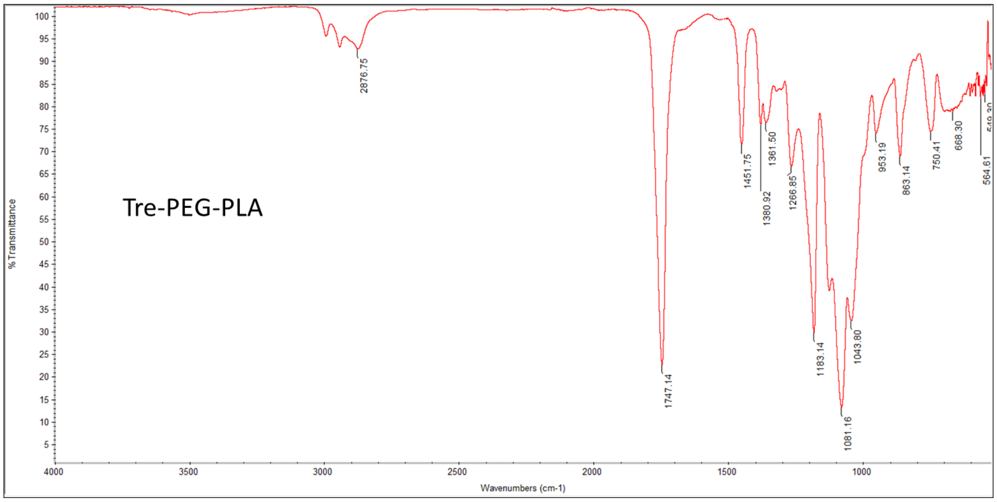

Figure S13. FT-IR spectrum of Tre-PEG-PLA.

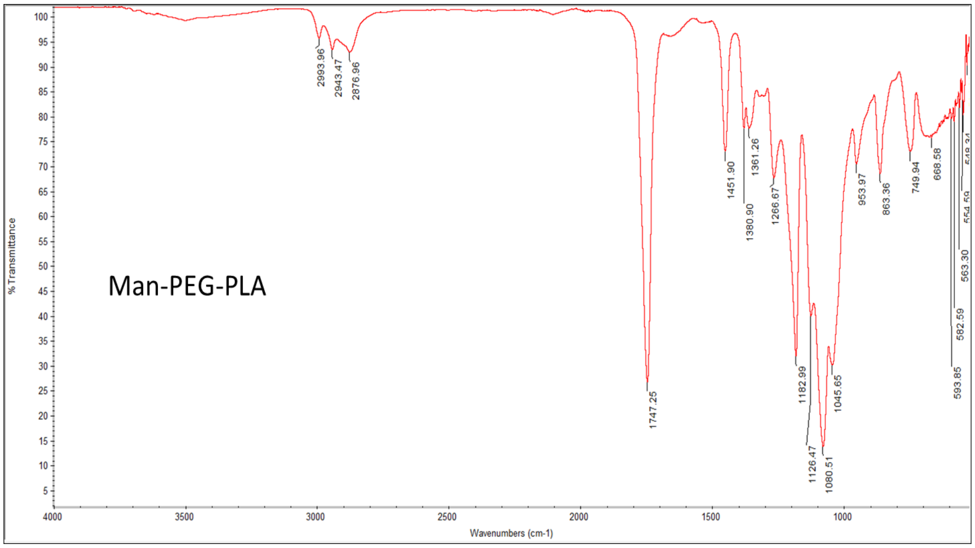

Figure S14. FT-IR spectrum of Man-PEG-PLA

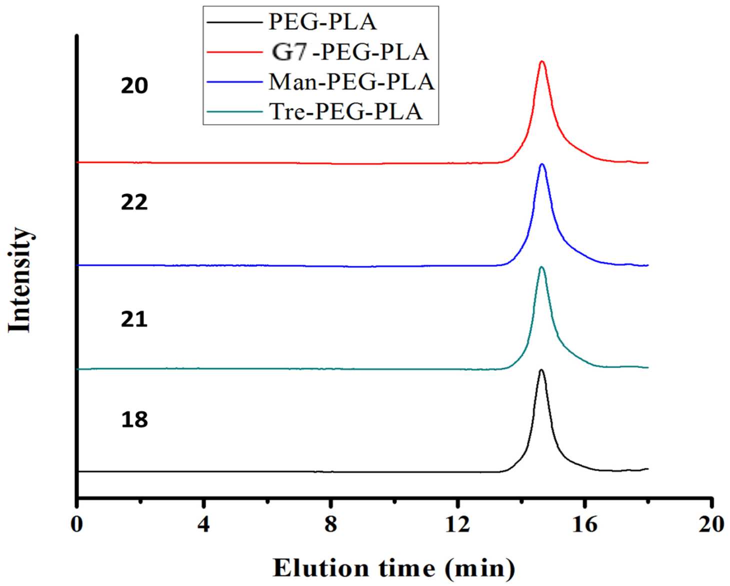

Figure S15. GPC traces of carbohydrate-PEG-PLA polymers (in DMF).

References

1. Farkas, E.; Jánossy, L.; Harangi, J.; Kandra, L.; Lipták, A., Synthesis of Chromogenic Substrates of α-amylases on a Cyclodextrin Basis. *Carbohydr. Res.* **1997,** *303* (4), 407-415.
2. Marmuse, L.; Nepogodiev, S. A.; Field, R. A., "Click Chemistry" en Route to Pseudo-starch. *Org. Biomol. Chem.* **2005,** *3* (12), 2225-2227.
3. Hayes, W.; Osborn, H. M. I.; Osborne, S. D.; Rastall, R. A.; Romagnoli, B., One-pot synthesis of multivalent arrays of mannose mono- and disaccharides. *Tetrahedron* **2003,** *59* (40), 7983-7996.
4. Richardson, A. C.; Tarelli, E., Chemical Modification of Trehalose. Part IX. The Monobenzylidene Acetal. *J. Chem. Soc. C* **1971,** (0), 3733-3735.
5. Backus, K. M.; Boshoff, H. I.; Barry, C. S.; Boutureira, O.; Patel, M. K.; D'Hooge, F.; Lee, S. S.; Via, L. E.; Tahlan, K.; Barry, C. E.; Davis, B. G., Uptake of Nnnatural Trehalose Analogs as a Reporter for Mycobacterium Tuberculosis. *Nat. Chem. Biol.* **2011,** *7* (4), 228-235.
